# Later-life coping in wartime captivity: a qualitative study of older civilian hostage returnees

**DOI:** 10.1093/geronb/gbag146

**Published:** 2026-07-21

**Authors:** Moshe Bensimon, Amit Shrira, Yuval Palgi, Yaakov Hoffman

**Affiliations:** Department of Criminology, Bar-Ilan University, Ramat Gan, Israel; Department of Social and Health Sciences, Bar-Ilan University, Ramat Gan, Israel; Department of Gerontology, University of Haifa, Haifa, Israel; Department of Social and Health Sciences, Bar-Ilan University, Ramat Gan, Israel; (Social Sciences Section)

**Keywords:** Hostage taking, Coping, Resilience, Qualitative research, Gerontological theory

## Abstract

**Objectives:**

Older civilian hostages are often framed as vulnerable, yet little age-specific evidence describes how they cope during captivity. This qualitative study examined coping strategies among Israeli older adults abducted by Hamas and interpreted findings through gerontological lenses.

**Methods:**

Semi-structured interviews were conducted with 13 older adults (mean age = 79.15; 12 women) approximately 1 year after release. Data were analyzed using a phenomenological approach to capture the lived experience of captivity.

**Results:**

6 themes captured coping as a flexible configuration of practices. Participants described emotion regulation through shutdown, crying, and mental quieting. Agency was preserved through bodily self-care, time structuring, writing, and cognitive tasks. Internal occupation through music, games, and autobiographical imagination supported continuity. When held with others, participants sustained interpersonal bonds through conversation, humor, storytelling, imaginative travel, mutual care, and leadership. Meaning-making frameworks supported hope and sustained expectations of return. Coping was not uniformly adaptive: some responses reflected ambivalent meaning-making and pointed to limits of coping, including reframing deprivation as shared suffering, guilt, perceived abandonment, and eroded trust.

**Discussion:**

Findings extend the literature by showing that later-life coping under external constraint involves preserving identity, relying on meaningful bonds, and shifting toward internal control. Results highlight vulnerability and strength, suggesting coping is active, socially embedded, and shaped by age-related meanings, while also revealing moral strain and ruptures in trust. Specifically, life stories and internal cognitive resources serve as survival mechanisms, transforming passive endurance into active engagement. These findings emphasize age-sensitive frameworks for understanding trauma and resilience among older populations in extreme situations.

Kidnapping and hostage taking occur across criminal, political, and terrorist contexts, yet systematic follow-up remains methodologically difficult. The evidence base relies heavily on clinical syntheses and autobiographical accounts rather than standardized datasets (Alexander & Klein, 2009). Captivity is conceptualized as a severe traumatic exposure with substantial psychological consequences (Grossman et al., 2019; Hoffman et al., 2018) that may extend well beyond release (Itzhaky & Mann, 2025). Hoffman et al. (2018) further specify prolonged captivity as a traumatic condition in which one’s destiny is under another’s control and escape is unfeasible. Within this literature, coping is described as heterogeneous and dynamic. Under acute threat, it may involve attentional narrowing and psychic numbing, while over time, it includes deliberate efforts to preserve functioning through mental activity, distraction, routine, and self-care (Alexander & Klein, 2010). Despite these insights, age-stratified evidence remains limited, in part because investigating acute trauma is ethically and practically difficult (Alexander & Klein, 2009). This gap also characterizes non-wartime contexts of kidnapping and hostage-taking, where existing literature has largely focused on mixed-age survivor populations, clinical effects, post-release adjustment, and general coping strategies rather than on older adults specifically (Alexander & Klein, 2009, 2010). Thus, although this broader literature provides useful background for understanding captivity-related trauma and coping, it does not offer a direct comparative framework for examining later-life coping in captivity.

The October 7, 2023, abductions in Israel created an unusually documented civilian case with a substantial representation of older adults. Among the 251 hostages, 13% were aged 65 and older (Clarfield & Levine, 2024). Media-based testimonies reveal several coping mechanisms, for example, hope anchored in family, adherence to routines, imagination, and interpersonal strategies such as supportive ties. Older abductees were more often portrayed as assuming leadership and caregiving roles (Levkovich et al., 2025; Shelef & Spector Mersel, 2025). These studies rely on public narratives rather than researcher-led interviews, underscoring the need for systematic qualitative inquiry.

Given the limited direct evidence specific to older adults, four gerontological theories are narrowly reviewed as interpretive frameworks, and their relevance to late-life captivity is highlighted. The Continuity Theory (Atchley, 1989) suggests that, when adapting to change, middle-aged and older adults tend to preserve familiar internal and external structures by drawing on strategies linked to their past experiences of themselves and their social worlds. Continuity is not defined as the absence of change, but as the maintenance of coherent patterns of identity, roles, relationships, activities, and meaning across changing circumstances. In captivity, this framework may help interpret how older adults draw on familiar habits, autobiographical resources, role identities, and relational patterns to sustain psychological coherence despite extreme disruption and loss of environmental control. Socioemotional Selectivity Theory (Carstensen et al., 1999) suggests that perceived time horizons shape motivational priorities, such that limited time leads individuals to become more selective in prioritizing emotionally meaningful goals, close relationships, and emotion regulation. Under radically uncertain and life-threatening captivity conditions, this perspective may help interpret participants’ orientation toward meaningful bonds, imagined reunion, and efforts to regulate emotional experience. The Life Span Theory of Control (Heckhausen & Schulz, 1995) distinguishes between primary control, aimed at changing the external environment, and secondary control, aimed at adjusting internal states in response to constrained or unattainable external control. Because direct control over the environment was severely restricted in captivity conditions, this theory may help explain how older adults preserved a sense of efficacy through attentional regulation, emotional self-management, and short-horizon goals. Finally, the Selection, Optimization, and Compensation model (Baltes & Baltes, 1990) frames adaptation as selecting attainable goals, optimizing available resources, and compensating for losses through alternative means. Applied to captivity, this model may help interpret how older adults allocate limited physical, cognitive, and relational resources toward manageable tasks, such as maintaining routines, caring for others, preserving bodily functioning, or sustaining mental occupation.

The present study focuses on older adult members living in Kibbutz, a rural community that emphasizes cooperative and egalitarian ideals and collective responsibility for members’ welfare (Leviatan, 1999). This social organization has also been associated with comparatively favorable functional outcomes among older members relative to other Israeli localities (Walter-Ginzburg et al., 2004). Because older adults in the current context were all Kibbutz members, this context provides an important backdrop for examining later life coping during captivity.

## The present study

The emerging literature on wartime captivity has provided important descriptive foundations, yet important gaps remain. Existing hostage and captivity literature has a limited empirical base and has primarily addressed coping among mixed-age hostage populations rather than among older adults specifically (Alexander & Klein, 2009, 2010). Similarly, recent accounts of October 7 civilian captivity have relied largely on publicly available media materials rather than direct researcher-led qualitative interviews. Shelef and Spector Mersel (2025) are particularly relevant to the present focus on older adults, as they analyzed broadcast interviews with 19 released civilian women and included a subgroup of six older women aged 63–85, five of whom were over 65. However, their study, like other recent work in this area, relied on publicly available media interviews rather than direct researcher-led qualitative interviews (Levkovich et al., 2025; Shelef & Spector Mersel, 2025). Against this background, the current study employs qualitative methods to examine how 13 Israeli older adults coped during captivity, drawing on direct, researcher-led interviews designed to elicit detailed, comparable accounts of experiences and coping efforts in captivity. By focusing specifically on older adults and interpreting their accounts through the four above gerontological perspectives, the study contributes age-specific, interview-based evidence on how later-life resources and vulnerabilities shape coping under extreme coercive conditions.

## Method

### Research approach

The study adopted a phenomenological orientation to qualitative inquiry, focusing on meanings individuals attribute to their lived experiences (Moustakas, 1994; Van Manen, 2014). Instead of testing predefined hypotheses, the inductive analysis remained open to directions emerging from participants’ accounts (Bogdan & Biklen, 1998). This approach enabled a nuanced, close exploration of their specific experiences in captivity.

### Participants

Thirteen Israeli older adults (*M*_age_ = 79.15 ± 6.79; Sex = 12 women; marital status, 30.8% married or living with a partner; socioeconomic status (SES) indexed by a single item ranging from 1 to 5, *M*_ses_ = 3.77 ± 1.01; education, 53.8% with academic degrees) residing in rural communities (Kibbutzim) in the Gaza envelope, who were kidnapped from their homes in October 2023 by Hamas militants, participated in the current study. Captivity duration was Md = 53 days. Among the 13 older hostages released, three were held alone throughout captivity, and one was held alone for most of the time. Four participants were held mostly with other adults, and five were held in mixed groups that included younger captives. Eight participants were held above ground, whereas five were held in underground tunnels. Participants were moved within Gaza between 0 and 4 times, with an average of 1.92 transitions. Eleven participants were exposed to some information during captivity, mainly through captors or media. Because all participants were older adults, systematic comparisons across age groups were not possible. However, studies of younger and mixed-age captives from the same war context reported similar captivity conditions, including transfers between locations, tunnel confinement, deprivation of basic needs, and restricted or manipulated access to information (Fennig et al., 2025; Katz et al., 2024; Shelef & Spector Mersel, 2025). Likewise, accounts from our participants held with captives younger than 65 did not point to obvious differences in food or information deprivation, though this observation is indirect.

### Procedure

The study was initiated after obtaining approval from the Bar-Ilan University Institutional Review Board (Approval No. 160524101). Participants were recruited with assistance from the Israeli Ministry of Welfare and Social Affairs. To maintain privacy, Ministry representatives initiated the initial contact with potential participants. Initial contact was established through social workers, followed by an approach tailored to their needs. Of the 251 Israelis taken into captivity, 33 were aged 65 and older. Among the 16 older adults who were released, one was not approached because of very poor health and died shortly thereafter, and two declined to take part, leaving 13 (86% of those approached) in the current sample. In two instances, the family member also spoke directly with the research team before giving consent. All 13 interviews were conducted at the participants’ current residences. Of the 13 interviews, seven were conducted by the lead researcher (M.B.), four by the fourth researcher (Y.H.), one by the third researcher (Y.P.), and one by a research assistant. At the time of the interviews, four participants were living in sheltered housing, two were staying with a family member, four were living in a temporary community, and three were residing in a vacant home that had been made available as temporary accommodation for released captives. Interviews were conducted on average 319.62 ± 43.15 days after their release (range: 246-389) amid the ongoing war. Each meeting began with signing an informed consent form and completing a brief demographic and quantitative questionnaire (approximately 1 hr in duration), which is not included in the present analysis (see: Hoffman et al., 2026). This first part was followed by a semi-structured qualitative interview after the participant was deemed sufficiently strong and willing to continue. Participants were reminded that they could pause or end the interview at any point and for any reason. The qualitative interviews lasted between 60 and 120 min. To ensure participants’ confidentiality, pseudonyms were assigned, and key contextual identifiers were altered to prevent deductive identification. ChatGPT 5.3 (OpenAI) was used solely to assist with language and style editing of selected portions of the manuscript. The authors entered text they had drafted to obtain suggestions regarding clarity, grammar, concision, and stylistic refinement. All analytic decisions, interpretations, and conclusions were made independently by the authors.

### Data collection and analysis

Data for the present study were collected through semi-structured, in-depth interviews guided by an open-ended interview guide (Cunningham et al., 2011). The key questions were: Describe the difficulties you had to cope with during captivity. What, if anything, helped you get through this period? How did you cope with uncertainty about your future? What was your daily routine, if any? Describe your interactions with your captors. Describe your interactions with other captives, if any were present.

The interviews were analyzed within a naturalistic phenomenological framework informed by Guba and Lincoln (1981). Analysis began with repeated readings of each transcript to gain an overall sense of the narrative and to note segments that related to coping with captivity. These meaning units were then coded and grouped into preliminary categories, which were compared across cases and, through an iterative process, refined into broader themes and subthemes. The lead researcher conducted the primary coding and thematic analysis for all 13 interviews.

In line with qualitative thematic analysis, the indication of “*n*” for each theme refers to the number of participants whose narratives contributed to that theme, either explicitly or implicitly, rather than to a statistical frequency. A participant was counted under a given theme whenever their account reflected that mode of coping in a meaningful way, even if the reference was brief or indirect. A summary of the themes, subthemes, and brief illustrative quotes is presented in Table 1.

**Table 1:** Qualitative themes, subthemes, and illustrative quotes regarding coping strategies among Israeli older adult captives (*N* = 13).

Theme	*n*	Subtheme	Brief quote/s
**1. Emotional regulation and coping with fear and uncertainty**	12	1.1 Coping with the possibility of death	“Eve and I talked about death… whoever loves me will love me.”; “I wasn’t very afraid of dying because of my (older) age.”
		1.2 Emotional disconnection and “shutter”	“Inside it was like a shutter.”; “My numbness… not to see, not to hear, not to feel.”
		1.3 Crying as emotional release and regulation	“I was crying there, really crying… and later (in captivity) I could also laugh about it.”; “I cried quietly, and it eased things for me.”
		1.4 Quasi-meditative self-calming and mental quieting	“I kept running a song through my head until I fell asleep.”; “It was like a kind of meditation… try to let the thoughts pass without catching them.”
**2. Maintaining control and agency**	12	2.1 Bodily self-treatment, physical activity, and use of prior bodily practices	“I did tai-chi… I used whatever tools I had in captivity.”; “I started moving my injured limbs by tapping on the wall repeatedly. Such behavior helped me recover from those injuries.”
		2.2 Constructing a daily structure and a calendar as anchors in time	“I made myself a calendar… and I wrote down all the birthdays”; “Every morning… Noam [another captive] would say what day it was, what date.”
		2.3 Daily writing and calculation as preservation of an organized thinking self	“I wrote many pages. One page every single day.”; “I took songs and turned them into numbers… little arithmetic puzzles.”
**3. Internal occupation and creativity**	11	3.1 Music and singing for oneself	“All the time, there was music playing in my head… it gave me a lot of quiet.”; “I sang all the time. It made me feel more alive.”
		3.2 Private mental games and autobiographical imagination as active self	“I made myself a TV program where one’s life is reviewed… I still had a life.”; “I played number games… so I wouldn’t think about anything else.”
**4. Interpersonal relationships as a mode of coping**	11	4.1 Assuming the role of an older adult: leadership, protection, and care	“I became the nurse… watching over the medicines… and I was also the representative.”; “I didn’t allow the young captives to be alone with one of the captors.”
		4.2 Group imagination, storytelling, and humor as mental escape	“We invented whole tours… it helped pass the time.”; “Come, I will tell you jokes.”
		4.3 Negotiating with captors while maintaining self-respect	“I mustn’t lower my gaze… that is what protects you.”; “…they agreed that at noon, I would… take whatever canned food I thought we needed.”
**5. Meaning-making and hope**	9	5.1 Drawing on the Holocaust, history, and life stories as a contextual resource	“…if children in the Holocaust… could write… then I should also try to write.”; “I had many lessons from those (Holocaust) books that I could use.”
		5.2 Hope and belief in eventual return	“And that is what kept us going, that every day that passes is one day less.”; “Every day that passes brings us closer to release.”
**6. Ambivalent meaning-making and limits of coping**	8	6.1 Reframing deprivation as shared suffering	“It isn’t that they are eating like kings and we are starving; it is all the same misery.”; “I couldn’t tell myself that we were the only one’s suffering… Everyone there is living in those bad conditions.”
		6.2 Moral strain and the erosion of trust	“I’m sorry you got dragged into this because of me.”; “… the Israeli government will leave us to die.”

*Note*. *n* represents the number of participants who mentioned the specific theme or subtheme. All participants were Israeli older adults released from captivity.

### Trustworthiness

Lincoln and Guba (1985) define trustworthiness by the extent to which researchers can be confident in their interpretations and can persuade readers that the findings are credible and worthy of consideration. In this study, peer debriefing was used as one strategy to enhance credibility. The researcher who led the analysis shared transcripts and preliminary coding for three interviews (interviews 1, 2, and 8) with the other three members of the research team. Each of the three other authors reviewed the material and provided written and oral feedback. This process also included meetings with the authors to discuss and refine groupings of thematic content. The feedback and team discussions informed subsequent analytic decisions and refinement of the thematic structure. This peer-debriefing process helped strengthen the credibility of the analysis.

## Results

The findings are organized into six themes. Themes 1 through 3 focus on individual-level experiences and actions described during captivity: (a) emotional regulation and coping with fear and uncertainty; (b) maintaining control and agency through bodily care and cognitive structuring of time; and (c) internal occupation and creativity. Themes 4 and 5 address relational and meaning-related dimensions: (d) interpersonal relationships, including mutual care, leadership, and interactions with captors; and (e) meaning-making and hope. Theme 6 captures coping accounts described as mixed or ambivalent in their effects, including normalization through shared misery and narratives of abandonment, guilt, or fractured trust.

### Theme 1: emotional regulation and coping with fear and uncertainty (*n *= 12)

#### Coping with the possibility of death

Confronting the possibility of not returning alive was central. Narratives combined practical considerations with existential reflections. Mina described conversations regarding her final wishes:

Eve and I talked about death. We knew what it would mean if I reached a worse condition, that they might take me to a (Gazan) hospital, and I wouldn’t come back. I told her to respect me, to respect what I wanted, that it is enough to plant a tree in the cemetery, that whoever loves me will love me, and that they do not need a body.

Natasha used her age as a psychological resource to shift concern toward younger captives:

I wasn’t very afraid of dying because of my (older) age. I told myself that I’m more worried about young captives than about myself since I’m already at such an (advanced) age. Thinking like that helped me. There is something in it that makes death feel a little less frightening.

#### Emotional disconnection and “shutter”

Some used deliberate numbing as an active protective stance to enable functioning. Esther linked her ability to function to emotional shutdown:

I think it helped me a lot that I didn’t think about the children, and I didn’t think about my family. Inside it was like a shutter that came down. I wasn’t afraid of anything, just a shutter, simply a shutter.

Rivka presented internal shutdown as a necessary choice for mental survival:

My numbness… at a certain point you had to shut yourself down—not to see, not to hear, not to feel, otherwise you would lose your mind. I sort of wrapped myself and went on automatic, because there was no other way to get through it.

#### Crying as emotional release and regulation

Alongside the shutdown, participants used crying as a cathartic release. Sara’s account illustrates how “really crying” enabled relief and later laughter:

I had moments of breakdown every Friday afternoon. Friday at five was always a crisis; you cannot imagine it. It was the time when I found out that at home, they were all around the table,^1^ and I wasn’t there. On a certain date, it was my birthday, and they (my captors) sang to me in Arabic, and I was crying there, really crying, and later (in captivity) I could also laugh about it.

Rachel framed silent crying as one of the only safe forms of emotional release in captivity, shaped by the constant need to remain quiet because the captors could hear and might enter at any moment:

For two days, I cried nonstop. I kept thinking about my mom. I told myself I couldn’t speak here because they could hear and might open the door. So, I cried quietly, and it eased things for me.

#### Quasi-meditative self-calming and mental quieting

Participants created inner space by deliberately quieting thoughts. Natasha used an intentional strategy to make stagnant time bearable:

I tried not to think too much, just to sit, stare, or close my eyes so that it would be quieter inside. In that way, I made it a little calmer and more relaxed for myself, as if I was putting a soft blanket over my thoughts. It was like a kind of meditation… try to let the thoughts pass without catching them. When I managed to do that, it was quieter, more peaceful inside.

Naomi described using repetitive mental focus to quiet her mind and make sleep possible:

I kept running a song through my head until I fell asleep. You must work your mind in order to fall asleep.

### Theme 2: maintaining control and agency (*n *= 12)

#### Bodily self-treatment, physical activity, and use of prior bodily practices

Some turned the injured body into a focus of self-initiated care. Rachel improvised repetitive movements that became purposeful action:

The pain in my body was terrible. […] I started moving my injured limbs by tapping on the wall repeatedly. It was like making music with my body. Such behavior helped me recover from those injuries, as my physician told me these actions restored function and movement.

Mina drew on a familiar bodily practice, tai-chi, to maintain a sense of routine:

I did tai-chi, something I had practiced before. I used whatever tools I had in captivity, whatever I learned. I just got up and did tai-chi in that room, and the captors thought I was crazy.

#### Constructing a daily structure and a calendar as anchors in time

Karin used a simple notebook as a calendar to link captivity to her family timeline:

First thing, when he [the guard] gave me a notebook, I made myself a calendar. That was the very first thing I did. I knew what the date was every day. And I wrote down all the birthdays of my children, my grandchildren, and my great-grandchildren.

Taylor stressed the importance of counting days to maintain temporal orientation:

About the time, every morning, at the beginning, Noam [another captive] would say what day it was, what date. How many days we had been in captivity. That way, I had a daily record.

#### Daily writing and calculation as preservation of an organized thinking self

Rachel used systematic daily writing to maintain her sense of “self” as a narrating person:

One of the women who guarded us came in. I told her I had seen a calendar diary in the children’s room. So, she said to me: “Start writing.” I wrote many pages. One page every single day. I would take the pages to the toilet with me and hide them in my clothes so they wouldn’t be found. In the end, they took a match and burned everything. But I have written every day.

Karin used arithmetic games and gematria^2^ to keep her mind busy:

I took songs and turned them into numbers. Every word I turned into its (Hebrew) numerical value, “gematria,” and I went through entire songs like that. I did this for maybe seventy songs. When I got tired of that, I moved on to doing little arithmetic puzzles, just to keep my mind busy.

### Theme 3: internal occupation and creativity (*n *= 11)

#### Music and singing for oneself

Dina used active singing to reconnect with earlier life roles and cultural identity:

I would go through all the songs in a row. I did the army troupe songs, then the Kibbutz songs, all the songs we used to sing. […] I sang all the time. It made me feel more alive.

Karin drew on classical pieces to create a sense of calm and continuity:

All the time, there was music playing in my head. All the time. Mostly classical music. It was there in the background, and it gave me a lot of quiet, like I was somewhere else for a moment, not only in that room.

#### Private mental games and autobiographical imagination as active self

Lucy used autobiographical imagination to reclaim her life story amid disruption:

I used my imagination. I made myself a TV program where one’s life is reviewed, as if they were telling the story of my life, and I was one of those people on the program. I went over all the periods and the important people. It helped me feel that I still had a life, that I wasn’t only what was happening there.

Esther invented a counting game to occupy her mind and protect herself from intrusive thoughts:

I played only with myself, so as not to lose my sanity. I played number games, such as counting backwards from 100, or counting but omitting numbers divisible by 6, and later from a hundred backwards. That is what I did all the time, so I wouldn’t think about anything else. Just this game, and that was it.

### Theme 4: interpersonal relationships as a mode of coping (*n *= 11)

#### Assuming the role of an older adult: leadership, protection, and care

Lifelong maturity allowed some to transform seniority into authority. Dina stabilized the group by assuming leadership:

I decided that wherever I would be, one thing I must do is help others who are there. If there is any way to help someone, then I’ll help. I took care of the food and handled all the small details that needed to be arranged. I became the nurse, the one watching over the medicines, and I was also the representative (of the captives with me), the one who spoke to the guards. In a way, I took on a leadership role there.

Taylor focused on protecting younger members, creating a buffer against the captors:

We were making sure nothing would happen to the young people at any time. I didn’t allow the young captives to be alone with one of the captors. I would always step in between them. […] I took on a kind of guard role as well, always looking around to see what was happening.

#### Group imagination, storytelling, and humor as mental escape

Group imagination, storytelling, and humor served as a form of mental escape that helped participants momentarily transcend physical confinement and help endure the monotony of captivity. Rivka recalled how shared laughter helped time pass:

After people finished giving their talks, one captive said: “Come, I will tell you jokes.” […] Afterwards, another captive decided that he would give us lectures about cinema, about films and directors, and we listened to him as if we were in a course.

Taylor described how shared imagination allowed the group to “travel” together:

We invented whole tours in our heads. One day we were in the US, at Niagara Falls; another day we would say: “Now we are in San Francisco” and we would talk about the place as if we were really there, going to the beach, walking around. It was all just from our minds, but it helped pass the time.

#### Negotiating with captors while maintaining self-respect

Naomi describes how she deliberately managed her relationship with her captor using eye contact and posture to shape how he perceived her and to preserve her dignity:

From the first moment, I understood that I mustn’t lower my gaze. I needed to look him in the eyes and create some kind of personal connection. That is what protects you. The more you make an interpersonal connection, the lower the chance that they will kill you. I also understood that I mustn’t show weakness. I became the point of contact for my group. I didn’t cry even once in front of them; I just looked them straight in the eyes. In a way, you want them to treat you, absurdly as it sounds, as an equal, with more self-respect, more respect.

Dina describes how she extended her caregiving role in captivity by speaking directly and confidently with her captors to negotiate practical arrangements and secure essential resources for the group:

I spoke with them very freely. I would tell them everything—what needed to be done and how to do it. In the end, they agreed that at noon, I would go over there and take whatever canned food I thought we needed. I was the one in charge of life there in that sense, making sure we had what we could have.

### Theme 5: meaning-making and hope (*n *= 9)

#### Drawing on the holocaust, history, and life stories as a contextual resource

Historical narratives provided a framework for survival. Rachel utilized the image of Anne Frank to maintain her drive to write:

I thought about children in the Holocaust. […] I told myself that if children in the Holocaust, with everything they went through, could write, then I should also try to write.

Naomi transformed lifelong learning into practical guidance for daily endurance:

I realized I had many lessons from those (Holocaust) books that I could use. Not to dwell on the past but to do exercises, to keep a daily routine, to know what the date is, and to try to sleep. I understood that I had to focus on survival and move forward only.

#### Hope and belief in eventual return

Taylor described how reframing time as progress toward release sustained spirits:

When a (brokered hostage) deal suddenly fell through because the prime minister did not sign, they would sink a bit into depression. Then Noam would say: “Okay, enough, this day is over, it is one day less in captivity.” And that is what kept us going, that every day that passes is one day less.

Naomi framed her certainty of release as a psychological anchor for herself and others:

I was sure we would get out of there safely. One hundred per cent. I told myself I don’t see a situation where I won’t get out of there. I knew that my certainty regarding that we would get out safely was what kept us going, and that is what I transmitted to the other captives. I told them all the time that every day that passes brings us closer to release.

### Theme 6: ambivalent meaning-making and limits of coping (*n* = 8)

#### Reframing deprivation as shared suffering

Some participants reduced the sense of personal injustice by framing deprivation as a shared condition. Abigail linked her own hunger to the broader hardship around her:

We lost seven or eight kilos because we ate less. […] The food wasn’t good, and there was never enough, but we got used to it. […] It isn’t that they are eating like kings and we are starving; it is all the same misery.

Similarly, Karin emphasized the wider conditions of suffering in Gaza:

I know that the children in Gaza live in the same terrible conditions that we lived in there, also with the food and with everything. So, I couldn’t tell myself that we were the only one’s suffering while they were fine. Everyone there is living in those bad conditions.

#### Moral strain and the erosion of trust

Other narratives reflected guilt, despair, and perceived abandonment. Sophie’s account conveyed moral strain, combining guilt toward her relatives with comfort in their being together:

When we arrived in Gaza, after more than twenty hours, I said to my relatives (who were with me in captivity): “I’m sorry if you were kidnapped because I chose to live next to the border, but if there is something I can be grateful for, it’s that I’m very happy you are together with me.” I told them: “I’m sorry you got dragged into this because of me, but it does me so much good that you are here with me.”

Mina’s narrative reflected eroded trust in political authorities:

When we talked, I said: “No one in the government really wants us to be released, because we don’t support this government. We come from the socialist left-wing Kibbutz movement, and the Israeli government will leave us to die.” No one replied. No one said: “You are wrong” or “You are correct”; my statement remained in the air unanswered.

## Discussion

This study extends the civilian captivity literature by providing interview-based accounts of coping among 13 Israeli older adults. Participants described emotional regulation as moving between confronting mortality and containing affect. This pattern aligns with clinical descriptions of early captivity responses, which may include attentional narrowing and dissociative-like phenomena under acute threat (Busuttil, 2008). It also accords with analyses of October 7 civilian testimony describing deliberate psychological disengagement as a means of maintaining day-to-day functioning in captivity (Levkovich et al., 2025; Shelef & Spector Mersel, 2025).

Agency was often preserved through controllable micro-domains such as bodily care, time structuring, and cognitive organization of activity. These accounts resonate with scholarship emphasizing efforts to impose order through routine, mental activity, and physical movement when feasible (Alexander & Klein, 2010). They also align with trauma theory that frames recovery from coercive trauma as involving the restoration of safety, control, and agency after experiences of domination and helplessness (Herman, 2015). In captivity, however, such restoration seems to have occurred through small embodied and cognitive gestures which were enacted under the continuing constraint of captivity.

Internal occupation supported self-continuity, enabling participants to remain active subjects under coercion. This is consistent with war captivity literature emphasizing the protective function of internal activities against the loss of control (Solomon et al., 1994). Such activities reflect what Frankl (1969, 1997) describes as the preservation of inner freedom through the intensification of intellectual and spiritual life. In Frankl’s terms, purpose can be sustained through creative values, mobilizing creativity from within as a buffer against dehumanization. In later life, these practices draw on accumulated autobiographical resources and familiar identity roles that support continuity in the face of abrupt rupture. Viewed in this way, imagination and creativity can support experiential reframing of adversity and the maintenance of agency under constraint (Bensimon, 2020, 2023).

When captives were held together, interpersonal processes became a central coping resource through connection, shared imagination, storytelling, humor, and mutual coordination. This pattern aligns with research on ex-captives’ testimony, highlighting supportive ties, emotional support, and resource sharing as interpersonal coping (Levkovich et al., 2025), and with media-based analyses emphasizing efforts to preserve togetherness under constraints (Shelef & Spector Mersel, 2025). More broadly, clinical scholarship suggests that group captivity can be protective, whereas solitary detention carries greater risk (Alexander & Klein, 2010). Earlier research among Vietnam War prisoners similarly reported being compelled into ongoing relationships and having to work through conflict with fellow prisoners, experiences some perceived as beneficial (Sledge et al., 1980). Because all participants were Kibbutz members, the prominence of mutual responsibility may be read as compatible with a communal backdrop described as emphasizing collective responsibility across the life course (Leviatan, 1999).

Meaning-making resources contextualized suffering within broader narratives and sustained hope, including drawing on Holocaust and historical frames, mobilizing life stories as a context for endurance, and using future-oriented belief in eventual return as a psychological anchor. Narrative gerontology links resilience with coherent life stories, compatible with participants’ efforts to sustain identity and meaning under coercion (Randall et al., 2015). The emphasis on life stories in the present findings may be interpreted in light of gerontological trauma frameworks that view life review as a means of integrating traumatic experiences into a coherent life narrative in older age. However, since previous research (e.g., Forstmeier et al., 2023) focused on structured clinical interventions, rather than on spontaneous coping in captivity, this connection should be understood as an interpretive application rather than direct empirical support.

At the same time, coping was not uniformly adaptive, and some accounts reflected ambivalent meaning-making and limits of coping rather than discrete strategies. Participants described reframing deprivation as a shared condition, which appeared to make their own suffering more bearable, while other accounts revealed moral strain and ruptures in trust, including guilt and perceived political abandonment. This pattern is consistent with scholarship indicating that captivity reactions involve guilt and anger and may unfold in phasic or prolonged courses (Alexander & Klein, 2010). Work on prolonged incarceration likewise points to complex adaptations during confinement, whose meanings may shift over time (Busuttil, 2008). Taken together, these convergences suggest that limits of coping become visible not only in overt breakdown, but also in more nuanced forms, such as interpretations that alleviate distress while revealing unresolved moral and relational burdens.

### Theoretical integration and conclusion: continuity and later life coping

This study suggests that older adults in captivity should not be framed solely as a vulnerable group. Rather, participants described drawing on a cumulative repertoire of habits, roles, and perspectives built over decades that served as usable coping tools under coercion. Because the study focused exclusively on older adults, these theoretical interpretations should not be read as comparative evidence that older hostages coped more effectively than younger hostages. Instead, they illuminate how later-life resources, including accumulated life experience, role continuity, emotion regulation, and meaningful bonds, were reflected in the accounts of this specific sample.

As noted in the introduction, the Continuity Theory (Atchley, 1989) helps interpret how participants preserved identity and psychological coherence through familiar routines, autobiographical reflection, role continuity, and long-standing relational patterns despite radical environmental disruption. Participants repeatedly described maintaining continuity through singing familiar songs, mentally revisiting life stories, structuring time, and sustaining caregiving and leadership roles. The emphasis on routine and mutual responsibility can also be read alongside descriptions of Kibbutz social organization that stress collective responsibility, while avoiding causal claims about captivity behavior (Leviatan, 1999). From this perspective, continuity functioned not as resistance to change but as a means of sustaining a coherent sense of self amid extreme disruption.

The Socioemotional Selectivity Theory (Carstensen et al., 1999) posits that when time horizons feel constrained, motivational priorities shift toward emotionally meaningful goals and close bonds, with emotion regulation becoming central. This perspective helps interpret how participants selectively anchored themselves in emotionally meaningful relationships, including imagined contact, future-oriented reunion, and relational connection within the group, as priorities that supported endurance under uncertainty. The findings suggest that emotional regulation became central to coping, reflected in efforts to maintain hope, avoid emotional collapse, preserve group cohesion, and focus attention on emotionally meaningful experiences.

The Life Span Theory of Control distinguishes between primary control, aimed at changing the environment, and secondary control processes that protect goal engagement when direct influence is limited (Heckhausen & Schulz, 1995). Here, coping often relied on secondary control processes such as emotional and cognitive self-regulation, attentional management, and short-term goal setting. These strategies appeared to preserve a sense of efficacy and psychological organization even when external control was severely restricted. The study contributes theoretically by showing how later-life control processes may be enacted hour by hour within the severely constrained conditions of captivity, where agency is preserved less through environmental change than through emotional and cognitive self-regulation.

The Selection, Optimization, and Compensation model (Baltes & Baltes, 1990) helps interpret how participants managed scarce resources under deprivation. Selection was reflected in narrowing attention to attainable and meaningful goals, such as maintaining bodily functioning, structuring time, or caring for others. Optimization appeared in repeated investment in these selected domains through micro-routines, cognitive activity, movement, and coordinated group roles. Compensation was evident when participants used alternative means to offset losses, such as relying on imagination, mental games, writing, humor, or shared caregiving when ordinary sources of control and stimulation were unavailable. Together, these patterns suggest pragmatic resource management under severely constrained external conditions.

In summary, coping in late-life captivity among older adults involved a flexible repertoire of practices supporting day-to-day endurance under coercion, including self-regulation, temporal and bodily routines, creative internal occupation, responsibility for others, and meaning-oriented frameworks. At the same time, it also comprised mechanisms that alleviated present distress while also revealing moral strain, mistrust, and the limits of what could be managed psychologically under captivity. Taken together, the findings extend gerontological theory by illustrating how continuity of self, selective emotional priorities, secondary control processes, and selection, optimization, and compensation can be enacted under conditions of near-total external constraint, and by highlighting coping as context-dependent and functionally shifting over time.

### Limitations and directions for future research

Several limitations should be considered. First, the sample comprised 13 Israeli older adults abducted from Kibbutzim; this specific context and the female-predominant sample may limit transferability and emphasize collective responsibility over other coping mechanisms. Second, the study relied on retrospective accounts collected after the first post-release year, and their memory recall may have been influenced by distress, public narratives, and post-captivity meaning-making. Longitudinal designs with repeated interviews could clarify how memories and the perceived functions of coping strategies change over the course of recovery. Third, participation required sufficient health and willingness, introducing survivorship and selection effects. Future research should broaden representation by comparing coping across age, gender, health status, captivity duration, and whether detention occurred alone or in groups. Comparative qualitative studies including both older and younger former hostages are needed to examine whether the coping patterns identified here reflect age-related resources, captivity conditions, cohort and community background, or broader human responses to prolonged coercive confinement. They should also assess whether captivity conditions differ by age, including location moves, tunnel confinement, food deprivation, and access to information. Such work would allow lifespan developmental perspectives to be examined more directly without inferring age-based differences from an older-only sample. Finally, the gerontological theories used were applied as interpretive lenses rather than as claims about age-related differences in coping. Future work should evaluate theory-informed hypotheses more directly, including whether socioemotional priorities, secondary control, and selective optimization processes predict coping patterns and longer-term adjustment.

### Implications for policy and practice

These findings have practical implications for preparedness and response systems supporting older adults exposed to captivity. Clinical scholarship emphasizes that captivity-related trauma requires sensitive management across the post-release period, including attention to delayed or intensifying reactions (Alexander & Klein, 2010; Busuttil, 2008). Age-responsive care should therefore include repeated assessment rather than reliance on immediate post-release symptom presentation alone. Literature on later-adulthood trauma reengagement suggests that trauma-related memories and distress may reemerge or intensify in later life as older adults encounter health decline, bereavement, role loss, increased dependency, and life review processes (Davison et al., 2016; Kaiser et al., 2023). Thus, early clinical impressions after release should not be treated as sufficient evidence of long-term psychological resolution.

For older hostage survivors, such care should combine trauma-informed support with gerontological attention to privacy, autonomy, family roles, community belonging, housing stability, and meaning-making. Clinically, this includes restoring daily structure, sleep patterns, bodily rehabilitation, and cognitive engagement, while monitoring emotional costs that may emerge over time, such as guilt, fractured trust, and moral strain. Because later-life losses or health changes may reactivate captivity-related distress, support should remain flexible, proactive, longitudinal, and sensitive to changing individual needs (Davison et al., 2016; Kaiser et al., 2023). Given that participants often described coping through responsibility for others and group coordination, follow-up should include relational support systems that recognize and support agency while helping survivors renegotiate responsibility. Such systems should also help prevent responsibility from becoming an additional burden. In the Kibbutz context, service planning can treat communal norms of solidarity and collective responsibility as contextual factors that may shape preferences for communal and group-based support, without assuming they are causal (Abramitsky, 2018; Leviatan, 1999). Policy should ensure coordinated longer-term pathways across health, welfare, and community systems, including proactive outreach for those at risk of functional decline.

## Author notes

1. For Israeli Jews, Friday night dinner is a unique custom where immediate and extended families dine together for a festive Friday night dinner.

2. Gematria refers to the numerical value of every Hebrew letter in the alphabet, so that one could transform words into numbers.

## Data Availability

This qualitative study used semi-structured in-depth interviews with older former hostages. To protect participants’ privacy and confidentiality, the raw interview data will not be publicly shared. Because of the small, highly identifiable sample and the sensitivity of the captivity narratives, de-identified transcripts cannot be made available for replication. The analytic procedures are described in the Method section, and additional details regarding the interview guide or coding process may be requested from the corresponding author, subject to ethical restrictions. The study was not preregistered. This study was approved by the Ethics Committee of Bar-Ilan University (approval number 160524101).
